# Transistor Properties of 2,7-Dialkyl-Substituted Phenanthro[2,1-*b*:7,8-*b*′]dithiophene

**DOI:** 10.1038/srep38535

**Published:** 2016-12-06

**Authors:** Yoshihiro Kubozono, Keita Hyodo, Shino Hamao, Yuma Shimo, Hiroki Mori, Yasushi Nishihara

**Affiliations:** 1 Research Laboratory for Surface Science, Okayama University, Okayama 700-8530, Japan; 2 Research Institute for Interdisciplinary Science, Okayama University, Okayama 700-8530, Japan; 3 Division of Earth, Life, and Molecular Sciences, Graduate School of Natural Science and Technology, Okayama University, Okayama 700-8530, Japan

## Abstract

A new phenacene-type molecule with five fused aromatic rings was synthesized: 2,7-didodecylphenanthro[2,1-*b*:7,8-*b*′]dithiophene ((C_12_H_25_)_2_-*i*-PDT), with two terminal thiophene rings. Field-effect transistors (FETs) using thin films of this molecule were fabricated using various gate dielectrics, showing p-channel normally-off FET properties with field-effect mobilities (*μ*) greater than 1 cm^2^ V^−1^ s^−1^. The highest *μ* value in the thin-film FETs fabricated in this study was 5.4 cm^2^ V^−1^ s^−1^, when a 150 nm-thick ZrO_2_ gate dielectric was used. This implies that (C_12_H_25_)_2_-*i*-PDT is very suitable for use in a transistor. Its good FET performance is fully discussed, based on electronic/topological properties and theoretical calculations.

Field-effect transistors (FETs) using thin films and single crystals of phenacene-type molecules (extended W-patterned structures of fused benzene rings) have been extensively studied using various gate dielectrics^1^,^2^,^3^,^4^,^5^,^6^,^7^,^8^,^9^,^10^,^11^,^12^,^13^,^14^,^15^,^16^,^17^,^18^. The highest field-effect mobility (*μ*) reported in these phenacene thin-film FETs is currently 21 cm^2^ V^−1^ s^−1^. This value was realized in a thin-film FET with 3,10-ditetradecylpicene ((C_14_H_29_)_2_-picene) (**1**), in which PbZr_0.52_Ti_0.48_O_3_ (PZT) was used as the gate dielectric^14^. In closely related studies, the highest *μ* value in a phenacene single-crystal FET is now 18 cm^2^ V^−1^ s^−1^. This was recorded in a [9]phenacene single-crystal FET, where ZrO_2_ was used as the gate dielectric^15^. It was recently found that the *μ* value in a phenacene-type single-crystal FET increases as the number of benzene rings increases, *i.e.*, more extended phenacene molecules are preferable for transistor applications owing to their greater intermolecular *π*-*π* overlap. These FET devices, using high-*k* gate dielectrics such as PZT and ZrO_2,_ showed p-channel normally-off FET characteristics under a low gate voltage (*V*_g_) enabling low-voltage operation. Thus, it has been found that phenacenes are very suitable molecules for use in FET devices. Furthermore, because of their deep highest occupied molecular orbital (HOMO) and the wide gap between their HOMO and their lowest unoccupied molecular orbital (LUMO), phenacene molecules are known to be more stable than acene-type molecules^9^, which is another advantage when using phenacene-type molecules in FET.

We have recently developed new phenacene-type molecules that include thiophene rings instead of solely benzene rings^19^,^20^,^21^. Phenanthro[1,2-*b*:8,7-*b*′]dithiophene (PDT) was first synthesized by our group^19^ and used in thin-film FET devices that showed p-channel normally-off FET properties with *μ* values as high as 10^−1^ cm^2^ V^−1^ s^−1^. This value was lower by one order of a magnitude than that in a picene thin-film FET^1^,^2^. Picene has the same number of rings as PDT, but it consists solely of benzene rings. Subsequently, six types of 2,9-dialkylated PDTs have been synthesized, with from 7 to 14 carbons in their alkyl chains, and used to fabricate thin-film FET devices^20^,^21^. All these FETs showed p-channel normally-off FET properties. The *μ* value in a 2,9-didodecylphenanthro[1,2-*b*:8,7-*b*′]dithiophene ((C_12_H_25_)_2_-PDT) (**2**) thin-film FET with an HfO_2_ gate dielectric was 2.2 cm^2^ V^−1^ s^−1^. The *μ* value of **2** was higher than those of other dialkyl-substituted PDTs.

In this paper, we propose a strategic approach to improving the *μ* value based on a recent study of picene and dinaphtho[1,2-*b*:2′,1′-*d*]chalcogenophenes^22^. This strategy follows from the fact that the HOMO level’s structure in **2** is different from that in **1,** which exhibits very high FET performance. This difference in electronic structure must cause significant differences in the *π*-*π* overlap between molecules, and thus the transfer integral between the molecules. Therefore, we considered how to introduce the thiophene rings into the PDT framework so that its HOMO resembles those of picene and **1**^14^. Based on theoretical calculations, we found that the 2,7-dialkylated phenanthro[2,1-*b*:7,8-*b*′]dithiophene ((C_n_H_2n+1_)_2_-*i*-PDT) had a HOMO with very similar characteristics to those in picene and **1** (*vide infra*). This isomer of PDT is phenanthro[2,1-*b*:7,8-*b*′]dithiophene (*i*-PDT), with the sulfur (S) atoms in its thiophene rings lying on the common axis of the two symmetrical phenyl rings, and *para* to the bond that joins them. Thus it was expected that the FET properties would be significantly improved owing to the similarity of this HOMO level’s characteristics to those of picene and **1**. In other words, since the HOMO’s wave functions in *i*-PDT resemble those of picene and **1**, the transfer integral should be enhanced by improved phase-matching between the wave functions of HOMO levels in neighboring molecules. This scenario is based on the band transport model for organic semiconductors. Also, the strength of hole-vibration coupling (or hole-phonon coupling (h-ph coupling)) between HOMO systems is essential for hole-hopping and polaron-related transport in organic semiconductors^23^. Although we have not yet evaluated the strength of h-ph coupling in the HOMO levels of *i*-PDT, we anticipated an increase in *μ* on the basis of the former model.

## Results

### Preparation of (C_12_H_25_)_2_-*i*-PDT 3

**Figure 1:**
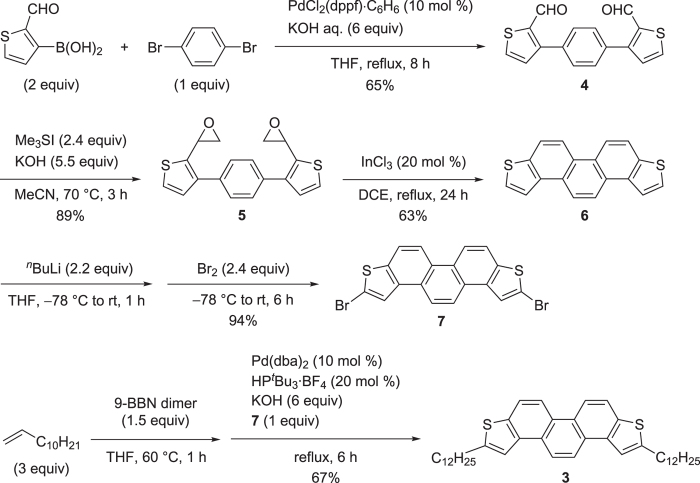
Synthetic procedure of (C_12_H_25_)_2_-*i*-PDT 3.

(C_12_H_25_)_2_-*i*-PDT **3** was synthesized in a similar manner to the synthesis of **2** as shown in Fig. 1 20. Experimental details are described in the Methods section and Supplementary Information. Palladium-catalysed Suzuki-Miyaura coupling of 2-formyl-3-thiopheneboronic acid and 1,4-dibromobenzene afforded the corresponding coupled product **4** in 65% yield. Subsequently, epoxidation of **4** gave the desired product **5** in 89% yield. We have been achieved the Friedel-Crafts type regioselective cycloaromatization of **5** with a catalytic amount of indium chloride afforded the cyclized product **6** as a sole product. Following this, bromination of *i*-PDT **6** with slight excess amount of butyllithium followed by addition of bromine afforded dibrominated *i*-PDT **7** in 94% yield. Finally, alkylation of **7** with alkylborane derived from hydroboration of terminal alkene and 9-BBN dimer afforded the desired product **3** in 67% yield. Obtained product **3** was further purified by sublimation twice in order to be applied to the OFET devices.

### Theoretical Calculations

**Figure 2:**
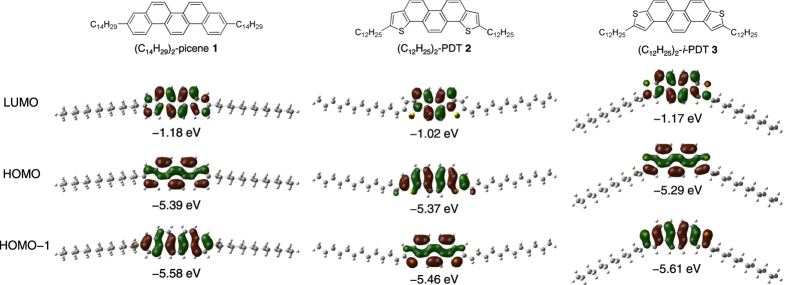
Molecular structures and orbitals of 1–3.

Figure 2 shows the molecular orbitals of **1**–**3**. As described previously, the HOMOs of **2** and **3** differ from each other. As seen from Fig. 2, the HOMO level of **2** decreases from −5.37 to −5.61 eV with a change in the position of the sulfur atoms, and becomes like HOMO-1 in **3**, while HOMO-1 of **2** rises from −5.46 to −5.29 eV, becoming like the HOMO in **3**. Clearly, the coefficients of the HOMO in **3** are now the same as those in **1**. The optimized geometry of **3** is shown in Fig. 2. All calculations were performed at the B3LYP/6–31 G(d) level using the Gaussian 09, Revision A. 02, program package^24^.

### Thin-Film Structure Analysis and Physicochemical Properties

**Figure 3:**
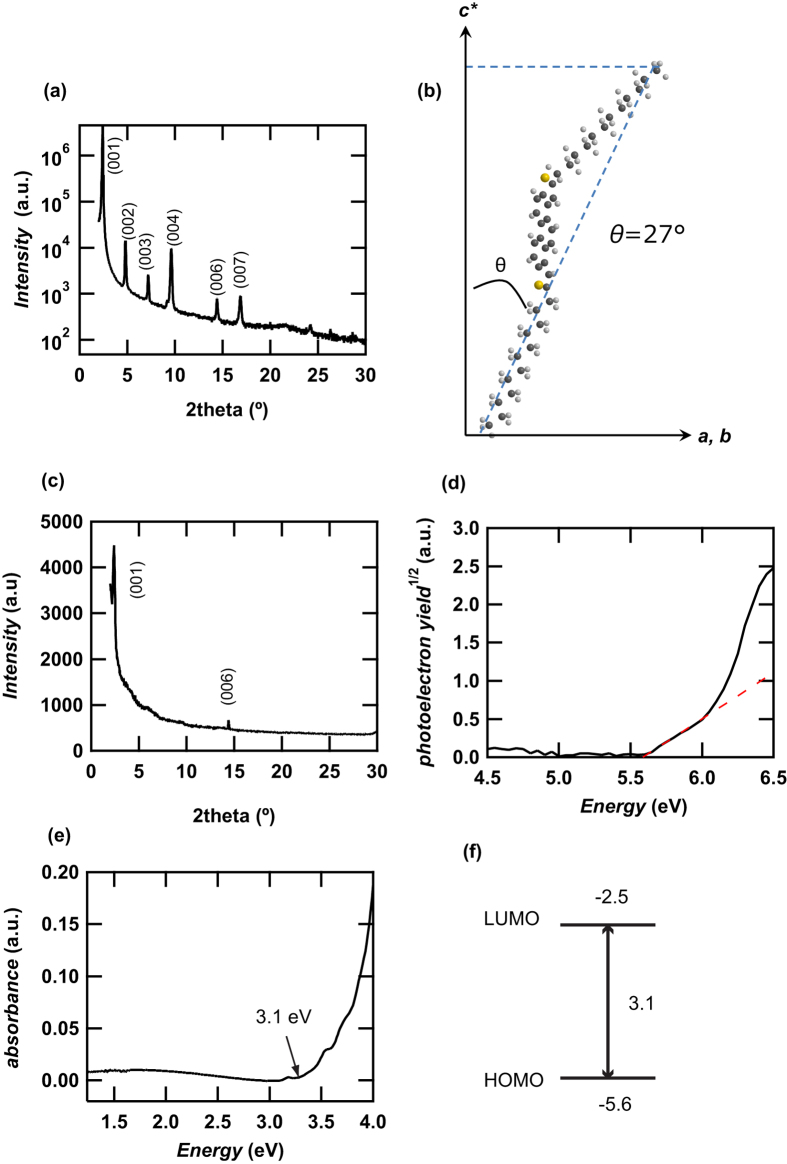
(**a**) XRD pattern of a thin film of **3** on SiO_2_ gate dielectric. (**b**) Schematic representation of orientation of **3** with respect to c*. (**c**) XRD pattern of a thin film of **3** on ZrO_2_ gate dielectric. (**d**) PYS spectrum, and (**e**) electronic absorption spectrum of thin films of **3** on SiO_2_ gate dielectric. (**f**) Energy diagram of thin films of **3** determined by PYS and electronic absorption spectra.

The out-of-plane XRD pattern of a thin film of **3** formed on an SiO_2_ gate dielectric is shown in Fig. 3(a); only *00 l* reflections were recorded to provide the plane spacing, *d*_001_, which means the *ab*-layers are stacked in parallel on the SiO_2_ gate dielectric. If the crystal structure of **3** is similar to phenacene’s^7^,^8^,^9^,^25^, the channel transports should be formed along the *ab*-plane. Therefore, the observation of *00 l* reflections is suitable for an FET device. The average *d*_001_, <*d*_001_>, evaluated from *00 l* reflections, corresponds to the reciprocal of lattice *c*, 1/|c*|. The <*d*_001_> value was evaluated to be 36.88(7) Å. If the optimized structure is a *trans*-form, the van der Waals length of the long axis of the molecule is expected to be 41.54 Å, indicating that the angle of inclination of **3** with respect to the c* axis is ca. 27° (Fig. 3(b)). This shows a strong similarity to phenacene molecules^4^,^5^,^9^,^26^. The out-of-plane XRD pattern of a thin film of **3** formed on a ZrO_2_ gate dielectric is shown in Fig. 3(c); only two peaks due to *001* and *006* reflections are observed, indicating the lower crystallinity. The *d*_001_ was estimated to be 37.0(2) Å, indicating that the angle of inclination of **3** with respect to the c* axis is ca. 27°. This is the same as that on SiO_2_ gate dielectric. However, the lowering of crystallinity was suggested in the thin film on ZrO_2_ gate dielectric.

**Figure 4:**
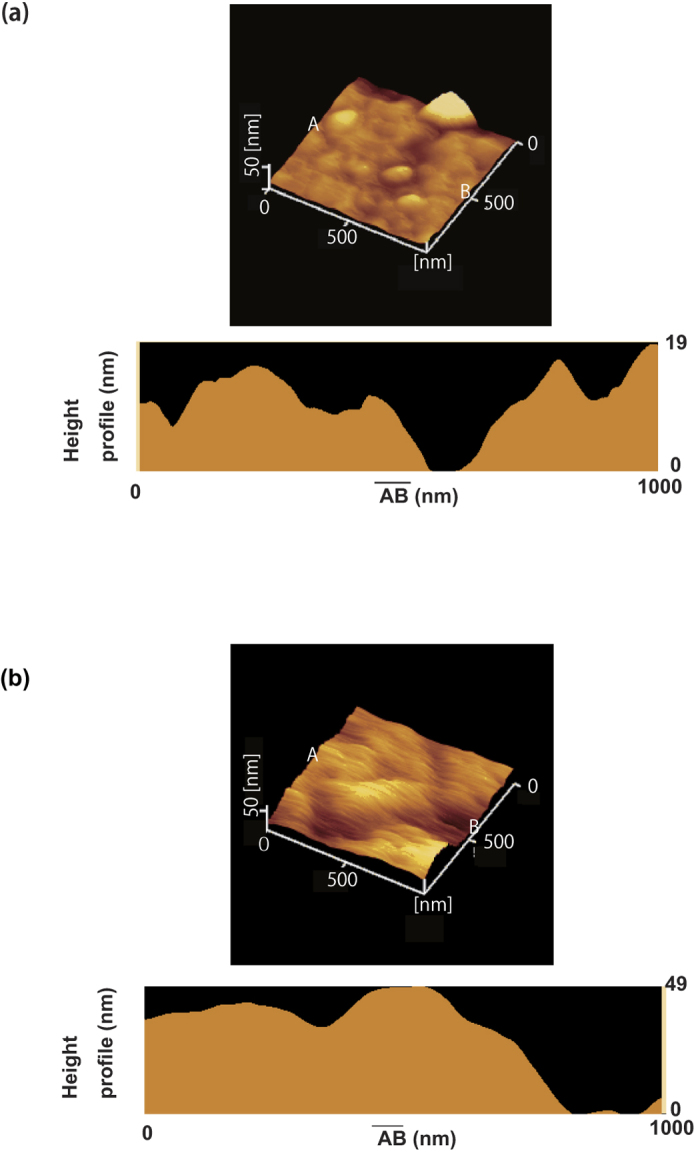
AFM images of thin films of **3** on (**a**) SiO_2_ and (**b**) ZrO_2_ gate dielectrics. Height-profile along the direction from A to B. The position of A and B are shown in the AFM images.

1 *μ*m × 1 *μ*m AFM images of a thin film of **3** on SiO_2_ and ZrO_2_ are shown in Fig. 4(a) and (b), respectively. Many grains with a diameter of 1 *μ*m were observed in the AFM image, and the root-mean-square (RMS) roughness for 60 nm-thick thin film was 5.3 nm on SiO_2_ and 10.1 nm on ZrO_2_, indicating that the roughness of thin film on ZrO_2_ is large. The value of 5.3 nm is almost the same as that of a 60 nm-thick thin film of **2** on SiO_2_ gate dielectric (see Fig. 4 of ref. 21). Therefore, the quality of thin film on ZrO_2_ is lower than that on SiO_2_ gate dielectric. The PYS and absorption spectra for the thin films of **3** on SiO_2_ are shown in Fig. 3(d) and (e), respectively. The onset of PYS was 5.6 eV, corresponding to its HOMO level (or the top edge of a valence band). The absorption spectrum showed the onset of the spectrum at 3.1 eV, which corresponds to the HOMO-LUMO gap. Figure 3(f) shows the energy diagram of **3**. The HOMO-LUMO gap is the same in both molecules, but the HOMO energy level of **3** (−5.6 eV), is higher than that of **2** (−5.8 eV). Because the Fermi level of Au is ca. −5.1 eV^27^, the HOMO of **3** is suitable for p-channel FET operation owing to the small energy-barrier between Au and the HOMO level. Such a notable alteration in HOMO energy level caused by changing the position of a sulfur atom in the PDT framework is quite significant, indicating a major change in the electronic structure.

### FET Characteristics

**Figure 5:**
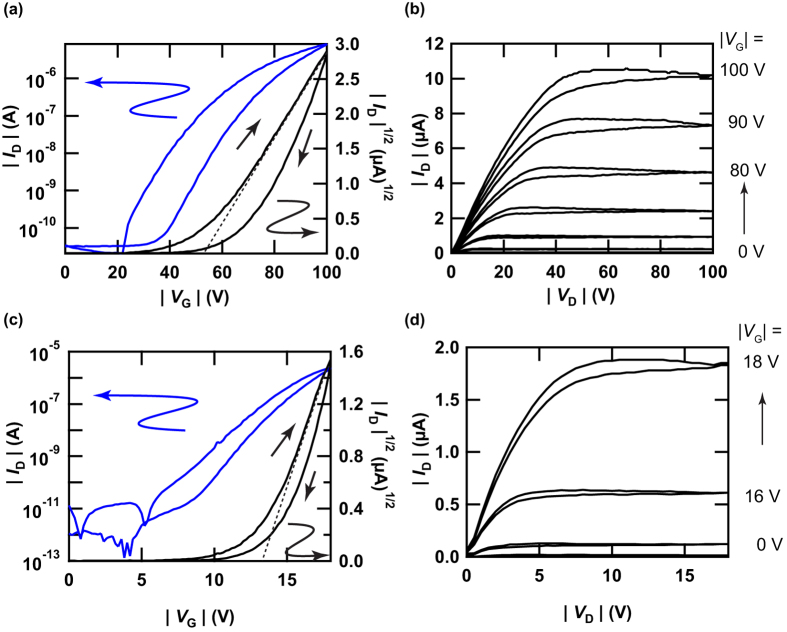
(**a**) Transfer and (**b**) output curves for a thin-film FET of **3** with SiO_2_ gate dielectric. (**c**) Transfer and (**d**) output curves for a thin-film FET of **3** with ZrO_2_ gate dielectric.

The transfer and output curves of a thin-film FET of **3** with a 400 nm-thick SiO_2_ gate dielectric are shown in Fig. 5(a) and (b), respectively. Typical p-channel FET properties were observed in the transfer and output curves. The values of *μ*, absolute threshold voltage (|*V*_th_|), on-off ratio, and subthreshold swing (*S* factor) were determined to be 8.0 × 10^−1^ cm^2^ V^−1^ s^−1^, 52 V, 4.1 × 10^5^, and 2.7 V decade^−1^, respectively, from the forward transfer curve at an absolute drain-voltage, (|*V*_D_|) of 100 V (saturation regime). Here, the channel width *W* and channel length *L* were 500 *μ*m and 450 *μ*m, respectively. Table S1 lists the FET parameters for ten FET devices with SiO_2_ gate dielectrics. The average *μ* (<*μ*>) was 5(2) × 10^−1^ cm^2^ V^−1^ s^−1^, smaller than that of **2**, which was 1.1(5) cm^2^ V^−1^ s^−1^.

Next, we changed the SiO_2_ gate dielectric to other gate dielectrics (ZrO_2_ and PZT) with high-*k* values. The transfer and output curves of a thin-film FET of **3** with a 50–150 nm thick ZrO_2_ gate dielectric are shown in Fig. 5(c) and (d), respectively; p-channel/low-voltage FET properties were observed. The *μ*, |*V*_th_|, on-off ratio, and *S* factor were determined to be 5.4 cm^2^ V^−1^ s^−1^, 7.8 V, 1.1 × 10^7^, and 0.8 V decade^−1^, respectively. This *μ* value is one of the highest among organic thin-film FETs. Table S2 lists the FET parameters determined for eleven FET devices with the ZrO_2_ gate dielectric fabricated in this study; the *W* and *L* were 500–1000 *μ*m and 100–450 *μ*m, respectively. The <*μ*> was 4.3(6) cm^2^ V^−1^ s^−1^, which is higher by a factor of three than the 1.8(6) cm^2^ V^−1^ s^−1^ of a thin-film FET of **2** with a ZrO_2_ gate dielectric. The <|*V*_th_|> was 10(2) V, which is the same as the 11.9(2) V of a thin-film FET of **2** with a ZrO_2_ gate dielectric^21^. Thus using a thin film of **3** helped improve FET performance.

Finally, we report the FET properties of a thin-film FET of **3** with a 150 nm-thick PZT gate dielectric. The FET provided p-channel normally-off properties, and the *μ* value was 5.6 cm^2^ V^−1^ s^−1^, with the same as that of a thin-film FET of **3** with a ZrO_2_ gate dielectric. The <*μ*> was estimated to be 4(1) cm^2^ V^−1^ s^−1^ from seven FET devices. FET parameters for seven FET devices with the PZT gate dielectric are listed in Table S3. Thus, molecule **3** can produce very good FET performance when a high-*k* gate dielectric is used.

## Discussion

The thin film FET of **3** formed on ZrO_2_ gate dielectric shows excellent FET properties with the <*μ*> value as high as 4.3(6) cm^2^ V^−1^ s^−1^. The value is higher than that, 5(2) × 10^−1^ cm^2 ^V^−1^ s^−1^, of the FET of thin film of **3** formed on SiO_2_ gate dielectric, and it is comparable to that, 4(1) cm^2^ V^−1^ s^−1^, of the FET of thin film of **3** formed on PZT gate dielectric. Because the crystallinity of thin film of **3** on ZrO_2_ is lower than that on SiO_2_ and the surface roughness of thin film on ZrO_2_ is large, the origin of excellent mobility of the thin film FET using ZrO_2_ gate dielectric may not be assigned to the quality of thin film. The origin is still unclear, but we must consider the facts that the surfaces of ZrO_2_ and PZT gate dielectrics are coated with 30–50 nm thick parylene, and that the surface of SiO_2_ gate dielectric is coated with hexamethyldisilazane (HMDS). The former surface is generally more hydrophobic than the latter surface. The hydrophobic surface of gate dielectric may produce the smooth hole-transport in the channel region which is located between active layer and the surface of gate dielectric.

Moreover, we must focus on the difference in capacitance between SiO_2_ and high-*k* (ZrO_2_/PZT) gate dielectrics. As described in the method section, the values of capacitances per area, *C*_o_’s, were five times higher for ZrO_2_ and PZT gate dielectrics than that for SiO_2_. It is well known that the gate dielectric with high *C*_o_ value provides the low field-effect mobility because of the pushing-effect (vertical electric field) of carriers against the interface^28^, in particular rough surface causes such a reduction of *μ*^29^. However, the observed high <*μ*> value in the FETs with thin films of **3** formed on ZrO_2_ and PZT gate dielectrics indicates the small roughness of the surface of gate dielectric which may be provided by the parylene-coating of gate dielectric. To sum up, the parylene-coating may be an origin of higher mobility in high-*k* gate dielectrics than that in SiO_2_ gate dielectric.

In this study, we found that a HOMO level can be controlled by a change as simple as altering the position of the sulfur atoms in the PDT framework. Molecule **3** was superior to **2** for FET application, experimentally verifying the significance of HOMO characteristics as a diagnostic indication of good electronic overlap, since **3** resembled **1**. In addition, the thin-film morphology of **3** was almost the same as that of **2**, showing that the high *μ* value in the FET using thin film of **3** does not originate from the extrinsic factor such as quality of thin film but from the intrinsic one (electronic overlap) caused from the molecular structure. These results do not just apply to the molecular design of phenacene-type molecules, but should also help to clarify the chemistry of *π*-network molecules, including thiophene rings. This useful alteration of electronic structure and FET performance, caused by isomerization that relocated a symmetrical pair of sulfur atoms in an extended *π*-network, suggests a potential avenue to be explored in the design of molecules suitable as functional materials in electronic devices.

## Methods

### Chemicals

Unless otherwise noted, materials obtained from commercial suppliers were used without further purification. 2-Formyl-3-thiopheneboronic acid (Aldrich), 1,4-dibromobenzene (TCI), 1-dodecene (TCI), and 9-borabicyclo[3.3.1]nonane dimer (Kanto Chemical) were used as received.

### Experimental details of synthesis of 3

The synthetic method for **3** reported in Results section is more fully described in this section, which included the actual experimental procedure.

To a solution of 2-formyl-3-thiopheneboronic acid (2.3 g, 15 mmol, 2 equiv) in THF (150 mL) in a 300 mL of 2-necked round-bottomed flask under an argon atmosphere were added PdCl_2_(dppf) · benzene (607 mg, 0.75 mmol, 10 mol %), 3 M aqueous KOH (15 mL, 48 mmol, 6 equiv) and 1,4-dibromobenzene (1.8 g, 7.5 mmol, 1 equiv) were added at room temperature. The resulting reaction mixture was stirred at 80 °C for 8 h, quenched with water (100 mL), and extracted with chloroform (300 mL x 3). The combined organic layers were washed with brine and dried over MgSO_4_. Filtration and evaporation afforded a pale yellow solid. Washing by ethyl acetate gave the titled product **4** (1.45 g, 4.9 mmol, 65%) as a yellow solid. R_*f*_ = 0.23 (hexane: ethyl acetate = 5:1). Mp = 252–254 °C. FT-IR (KBr, cm^−1^): 3094 (s), 3076 (s), 3044 (s), 2855 (s), 2822 (s), 1659 (w), 1427 (w), 1354 (m), 1202 (w), 895 (m), 853 (m), 820 (m), 754 (w), 675 (w). ^1^H NMR (400 MHz, CDCl_3_, rt): *δ* 7.29 (d, *J* = 4.8 Hz, 2 H), 7.61 (s, 4 H), 7.80 (d, *J* = 4.8 Hz, 2 H), 9.95 (s, 2 H); ^13^C{^1^H} NMR (150 MHz, CDCl_3_, rt): *δ* 127.5, 129.9, 130.5, 134.4, 138.9, 150.1, 183.8. Anal. Calcd for C_16_H_10_O_2_S_2_: C, 64.40; H, 3.38%. Found: C, 64.28; H, 3.32%. ^1^H and ^13^C NMR spectra are shown in Figure S1 of Supplementary Information.

To a solution of dialdehyde **4** (1.2 g, 4 mmol, 1 equiv) in anhydrous acetonitrile (80 mL) in a 200 mL of 2-necked round-bottomed flask under an argon atmosphere were added trimethylsulfonium iodide (2 g, 9.6 mmol, 2.4 equiv) and powdered KOH (1.2 g, 22 mmol, 5.5 equiv) at room temperature. The reaction mixture was stirred at 70 °C for 3 h, quenched with water (100 mL), and extracted with chloroform (300 mL x 2). The combined organic layers were washed with brine and dried over MgSO_4_. Filtration and evaporation afforded the titled product **5** (1.16 g, 3.6 mmol, 89%) as yellow solid. Mp = 179–180 °C. FT-IR (KBr, cm^−1^): 3098 (s), 3076 (s), 3048 (s), 2986 (s), 1346 (m), 1246 (s), 968 (m), 840 (w), 741 (w), 662 (s). ^1^H NMR (300 MHz, CDCl_3_, rt): *δ* 3.12–3.13 (m, 2 H), 3.26 (dd, *J* = 5.1, 0.9 Hz, 2 H), 4.17 (dd, *J* = 3.9, 1.2 Hz, 2 H), 7.16 (d, *J* = 5.4 Hz, 2 H), 7.30 (d, *J* = 5.1 Hz, 2 H), 7.59 (s, 4 H); ^13^C{^1^H} NMR (150 MHz, CDCl_3_, rt): *δ* 48.8, 51.9, 124.3, 128.9, 129.3, 134.6, 135.9, 135.9, 141.9, 141.9. HRMS (EI^+^) Calcd for C_18_H_14_O_2_S_2_: 326.0435. Found: 326.0417. ^1^H and ^13^C NMR spectra are shown in Figure S2 of Supplementary Information.

To a solution of epoxide **5** (261 mg, 0.8 mmol, 1 equiv) in anhydrous 1,2-dichloroethane (40 mL) in a 50 mL of Schlenk tube equipped with a magnetic stir bar under an argon atmosphere were added indium chloride (35 mg, 0.16 mmol, 20 mol %) at room temperature. The reaction mixture was stirred at 100 °C for 24 h, quenched with water (5 mL) and poured into MeOH, which caused precipitation of pale-yellow solid. The suspension was filtered, and the solid was dried under vacuum afforded the titled product **6** (188 mg, 0.5 mmol, 63%) as a pale yellow solid. R_*f*_ = 0.42 (hexane: chloroform = 4:1). Mp > 270 °C. FT-IR (KBr, cm^−1^): 3102 (s), 3080 (s), 3067 (s),1385 (s), 1315 (s), 1194 (m), 826 (m), 797 (w), 691 (w), 592 (s). ^1^H NMR (300 MHz, CDCl_3_, rt): *δ* 7.66 (d, *J* = 5.1 Hz 2 H), 8.09–8.15 (m, 4 H), 8.50 (s, 2 H), 8.72 (d, *J* = 9 Hz, 2 H); ^13^C{^1^H} NMR (150 MHz, CDCl_3_, rt) was not obtained due to the poor solubility. Anal. Calcd for C_18_H_10_S_2_: C, 74.45; H, 3.47%. Found: C, 74.12; H, 3.44%. ^1^H NMR spectrum is shown in Figure S3 of Supplementary Information.

To a solution of *i*-PDT **6** (145 mg, 0.5 mmol, 1 equiv) in anhydrous THF (15 mL) in a 20 mL of Schlenk tube equipped with magnetic stir bar under an argon atmosphere were cooled at −78 °C, and then *n*-butyllithium (1.6 M in hexane, 690 *μ*L, 1.1 mmol, 2.2 equiv) was added dropwise. After being stirring for 1 h at room temperature, the mixture was cooled to −78 °C and bromine (62 *μ*L, 1.2 mmol, 2.4 equiv) was added dropwise. The reaction was stirred overnight at room temperature, quenched with water (5 mL) and poured into MeOH, which caused precipitation of pale yellow solid. The suspension was filtered, and the solid was dried under vacuum afforded the titled product **7** (210 mg, 0.47 mmol, 94%) as a pale yellow solid. Mp > 270 °C. FT-IR (KBr, cm^−1^): 3084 (s), 3061 (s), 2928 (s), 2862 (s), 1516 (s), 1485 (m), 1190 (m), 951 (m), 881 (m), 800 (w). ^1^H NMR (600 MHz, CDCl_3_, rt): *δ* 7.97 (d, *J* = 9 Hz, 2 H), 8.04 (s, 2 H), 8.32 (s, 2 H), 8.65 (d, *J* = 9 Hz, 2 H); ^13^C{^1^H} NMR (150 MHz, CDCl_3_, rt) was not obtained due to the poor solubility. HRMS (EI^+^) Calcd for C_18_H_8_Br_2_S_2_: 445.8434. Found: 445.8440. ^1^H NMR spectrum is shown in Figure S4 of Supplementary Information.

According to a synthetic procedure in the literature^30^, this new compound was prepared. To a solution of 1-dodecene (126 mg, 0.75 mmol, 3 equiv) in anhydrous THF (5 mL) in a 20 mL Schlenk under argon was added 9-BBN dimer (92 mg, 0.4 mmol, 1.5 equiv) at room temperature. The reaction mixture was stirred at 60 °C for 1 h, then cooled at room temperature and added Pd(dba)_2_ (14.4 mg, 0.03 mmol, 10 mol %), [HP*t*-Bu_3_]BF_4_ (14.4 mg, 0.06 mmol, 20 mol %), powdered KOH (196 mg, 1.5 mmol, 6 equiv), and compound **7** (112 mg, 0.25 mmol, 1 equiv) were added at room temperature. The reaction mixture was stirred at 85 °C for 6 h, quenched with water (10 mL), and extracted with chloroform (30 mL x 2). The combined organic layers were washed with brine and dried over MgSO_4_. Filtration and evaporation afforded a brown solid. Column chromatography on silica gel (hexane: chloroform = 2:1) gave titled product **3** (105 mg, 0.17 mmol, 67%) as a pale yellow solid, which was further purified by sublimation to give analytically pure samples as white solid. R_*f*_ = 0.79 (hexane: chloroform = 2:1). Mp = 220–221 °C. FT-IR (KBr, cm^−1^): 2957 (m), 2920 (w), 2874 (m), 2851 (w), 1468 (m), 1198 (m), 822 (m), 799 (w). ^1^H NMR (600 MHz, CDCl_3_, rt): *δ* 0.88 (t, *J* = 7.2 Hz, 6 H), 1.20–1.34 (m, 28 H), 1.35–1.40 (m, 4 H), 1.46 (quintet, *J* = 7.2 Hz, 4 H), 1.84 (quintet, *J* = 7.8 Hz, 4 H), 3.04 (t, *J* = 7.2 Hz, 4 H), 7.74 (s, 2 H), 8.00 (d, *J* = 8.4 Hz, 2 H), 8.37 (s, 2 H), 8.61 (d, *J* = 9 Hz, 2 H); ^13^C{^1^H} NMR (150 MHz, CDCl_3_, rt): *δ* 14.1, 22.7, 29.2, 29.4, 29.4, 29.6, 29.7, 31.1, 31.5, 31.9, 118.8, 118.9, 120.9, 122.9, 126.5, 127.6, 136.7, 137.2, 147.3. HRMS (EI^+^) Calcd for C_42_H_58_S_2_: 626.3980. Found: 626.3956. ^1^H and ^13^C NMR spectra are shown in Figure S5 of Supplementary Information.

### Fabrication and characterization of FET Devices

60 nm-thick thin films of **3** were fabricated on various gate dielectrics (SiO_2_, ZrO_2_, and PZT) formed on Si substrates by thermal deposition below 10^−7^ Torr. Surfaces of the gate dielectrics were modified by hexamethyldisilazane (HMDS) for SiO_2_ and parylene for ZrO_2_ and PZT for hydrophobicity; a monolayer of HMDS was formed on SiO_2_, and 50 nm-thick parylene was coated on PZT and ZrO_2_. The substrate was maintained at room temperature during thermal deposition. 50 nm-thick gold (Au) source/drain electrodes were formed on the thin film of **3** by thermal deposition below 10^−7^ Torr. A 3 nm-thick layer of 2,3,5,6-tetrafluoro-7,7,8,8-tetracyanoquinodimethane (F_4_TCNQ) was inserted between Au electrodes and the thin film to realize smooth carrier accumulation, or to reduce contact resistance. Thus a top-contact bottom-gate type FET device was fabricated with a thin film of **3** as an active layer. The FET properties were measured in two-terminal measurement mode using a semiconductor parameter analyzer (Agilent B1500A). The capacitances per area, *C*_o_’s, for the gate dielectrics were measured using a precision LCR meter (Agilent E4980A); the *C*_o_ values were 8.3 nF cm^−2^ for SiO_2_, 42 nF cm^−2^ for ZrO_2_, and 40 nF cm^−2^ for PZT, respectively.

### Instrumentation

All the reactions were carried out under an Ar atmosphere using standard Schlenk techniques. Glassware was dried in an oven (130 °C) and heated under reduced pressure before use. For thin layer chromatography (TLC) analyses throughout this work, Merck precoated TLC plates (silica gel 60 F_254_, 0.25 mm) were used. Silica gel column chromatography was carried out using Silica gel 60 (spherical, 40–100 *μ*m) from Kanto Chemicals Co., Ltd. NMR spectra (^1^H and ^13^C{^1^H}) were recorded on Varian INOVA-600 (600 MHz), Mercury-400 (400 MHz) and 300-NMR ASW (300 MHz) spectrometers. Chemical shifts (*δ*) are in parts per million relative to CDCl_3_ at 7.26 ppm for ^1^H and at 77.0 ppm for ^13^C{^1^H} NMR spectra. Infrared spectra were recorded on a Shimadzu IRPrestige-21 spectrophotometer and reported in wave numbers (cm^–1^). HRMS were determined on a JEOL JMS-700 MStation. Elemental analyses were carried out with a Perkin-Elmer 2400 CHN elemental analyzer. X-ray diffraction (XRD), atomic force microscopy (AFM), photoelectron yield spectroscopy (PYS), and electronic absorption were measured using a Smart Lab-Pro (Rigaku) with an X-ray wavelength of 1.5418 Å (Cu*Kα* source), an SPA 400-DFM (SII Nano Technology), a BIP-KV201AD PYS spectrometer (Bunko Keiki), and a JASCO V670 UV–vis spectrometer (JASCO), respectively.

## Additional Information

**How to cite this article**: Kubozono, Y. *et al*. Transistor Properties of 2,7-Dialkyl-Substituted Phenanthro[2,1-*b*:7,8-*b*′]dithiophene. *Sci. Rep.* **6**, 38535; doi: 10.1038/srep38535 (2016).

**Publisher's note:** Springer Nature remains neutral with regard to jurisdictional claims in published maps and institutional affiliations.

## Supplementary Material

Supplementary Information
