# Supplementary material for: Transistor Properties of 2,7-Dialkyl-Substituted Phenanthro[2,1-b:7,8-b′]dithiophene
Source: Sci Rep. 2016 Dec 6;6:38535. doi: 10.1038/srep38535 (PMC5138603; doi:10.1038/srep38535)
Supplement: Supplementary Information [file srep38535-s1.pdf]

Supplementary Information

Transistor Properties of 2,7-Dialkyl-Substituted Phenanthro[2,1-*b*:7,8-*b′*]dithiophene

Yoshihiro Kubozono,[†,‖] Keita Hyodo,[§] Shino Hamao,[†,‖]
Yuma Shimo,[†] Hiroki Mori,[‖] Yasushi Nishihara[*,‖]

[†]*Research Laboratory for Surface Science, Okayama University, Okayama 700-8530, Japan*
[‖]*Research Institute for Interdisciplinary Science, Okayama University, Okayama 700-8530, Japan*
[§]*Division of Earth, Life, and Molecular Sciences, Graduate School of Natural Science and Technology, Okayama University, Okayama 700-8530, Japan*

Phone: +81-86-251-7855
Fax: +81-86-251-7855
Email: ynishiha@okayama-u.ac.jp

Contents

1. Copies of ^{1}H and ^{13}C NMR Charts for the New Compounds S2–S6
2. Table S1. FET parameters of $(C_{12}H_{25})_2$-iPDT thin-film FETs with SiO_2 gate dielectrics. S7
3. Table S2. FET parameters of $(C_{12}H_{25})_2$-iPDT thin-film FETs with ZrO_2 gate dielectrics. S8
4. Table S3. FET parameters of $(C_{12}H_{25})_2$-iPDT thin-film FETs with PZT gate dielectrics. S9

1. Copies of ^{1}H and ^{13}C{^{1}H} NMR Charts for the New Compounds

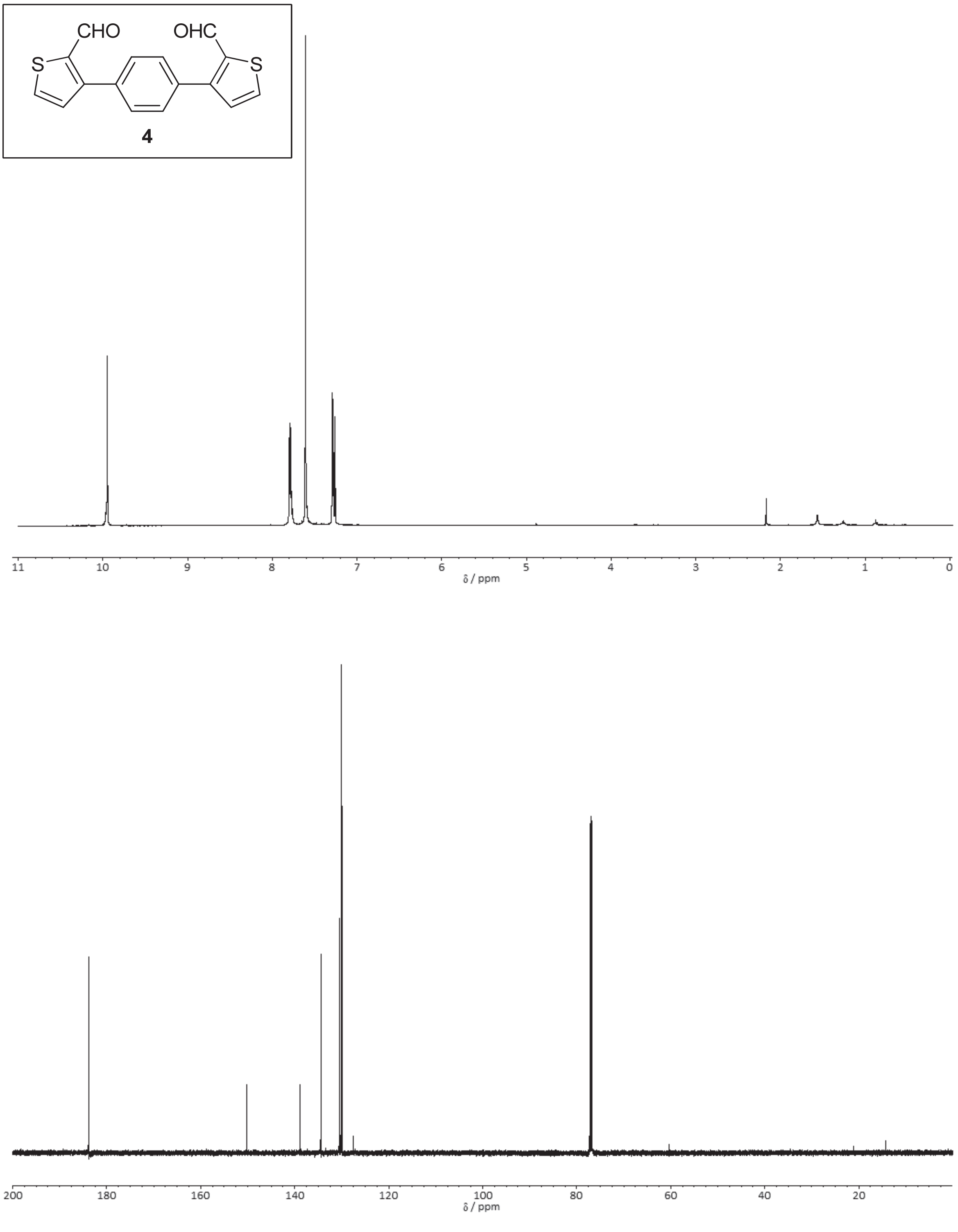

Figure S1. The ^{1}H and ^{13}C{^{1}H} NMR spectra of compound **4** (in $CDCl_3$).

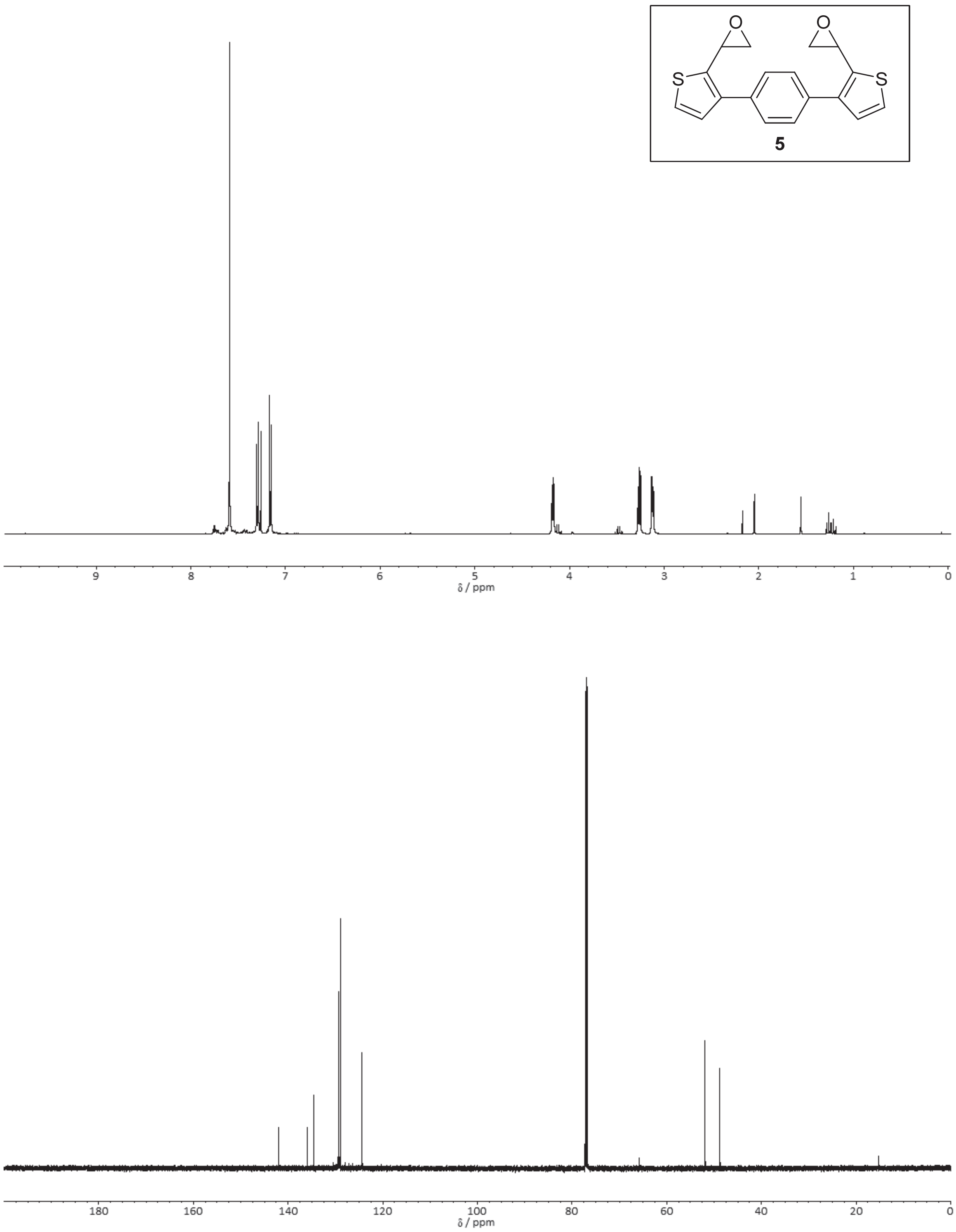

Figure S2. The ^{1}H and ^{13}C{^{1}H} NMR spectra of compound **5** (in $CDCl_3$).

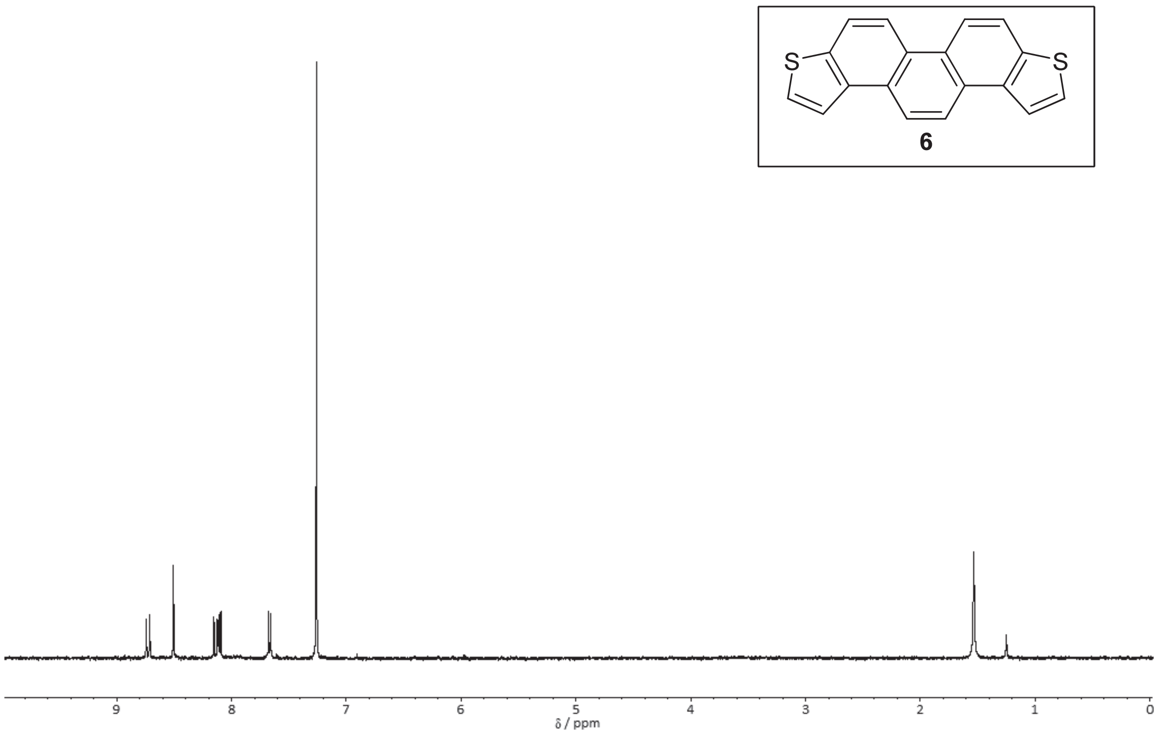

Figure S3. The ^{1}H NMR spectrum of compound **6** (in $CDCl_3$).

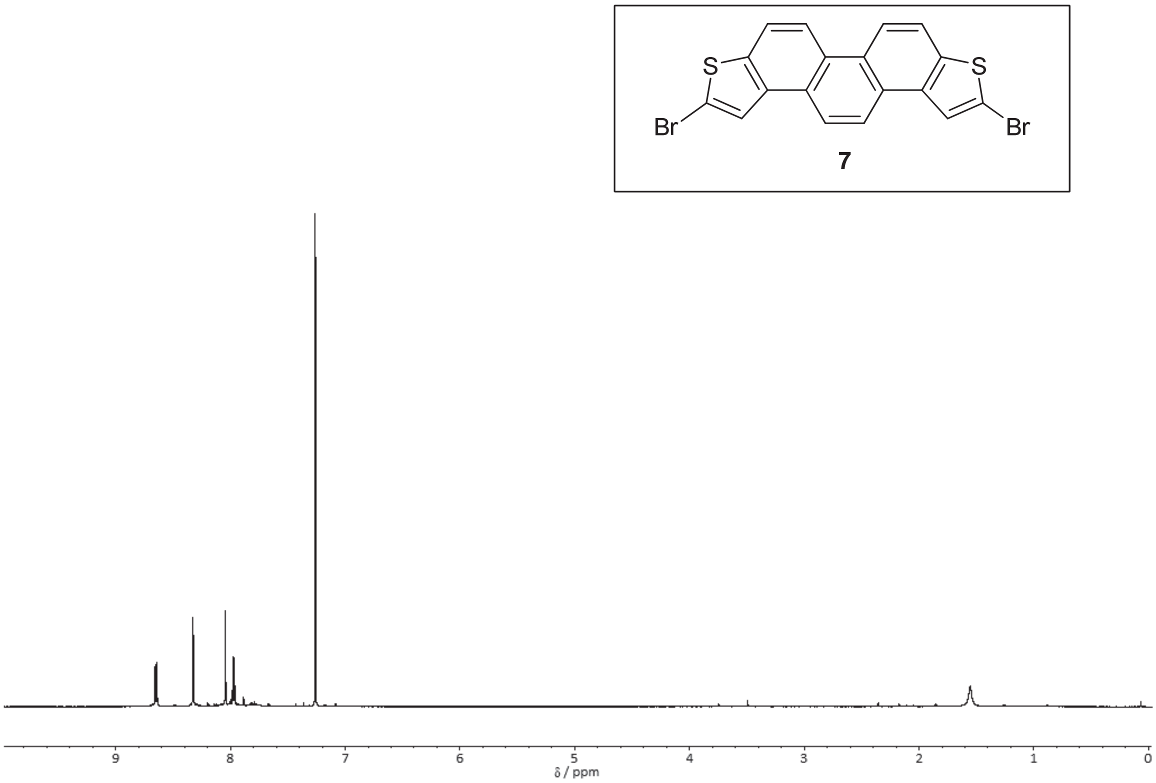

Figure S4. The ^{1}H NMR spectrum of compound **7** (in $CDCl_3$).

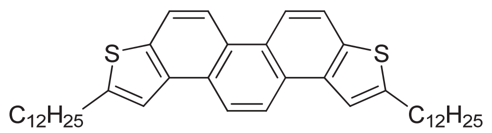

3

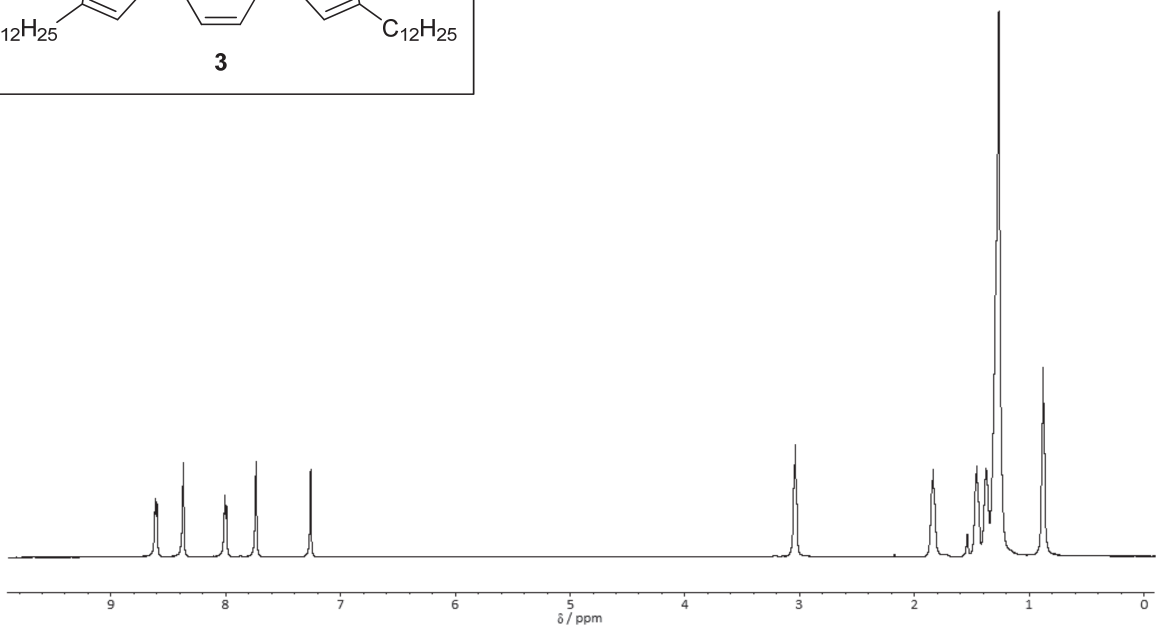

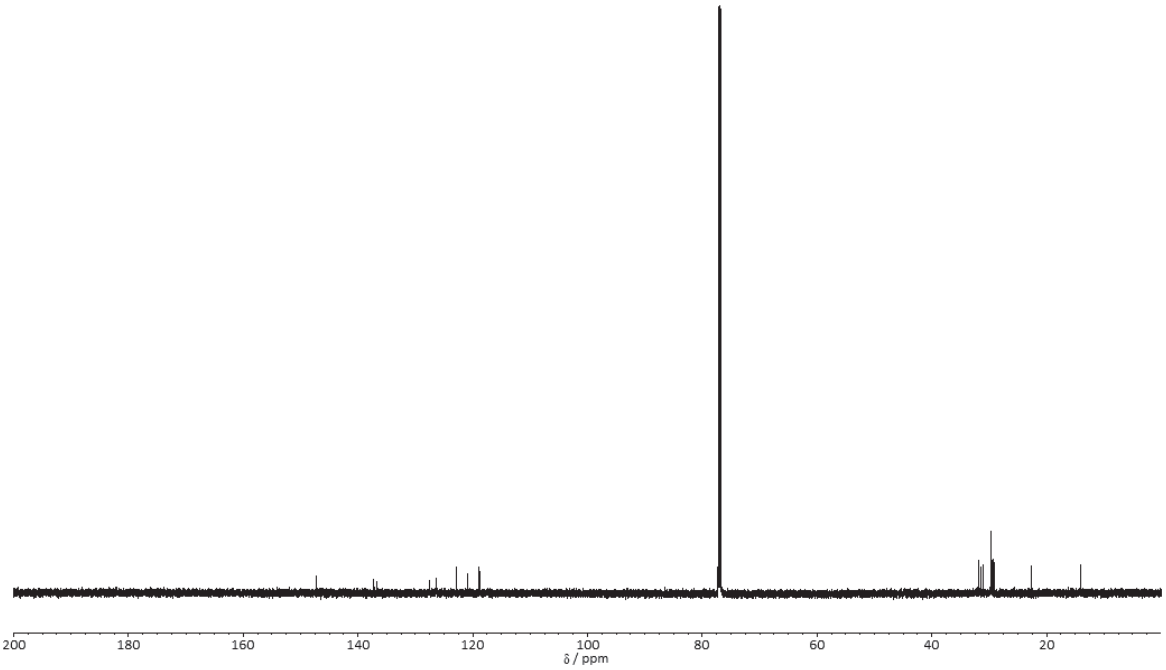

Figure S5. The ^{1}H and $^{13}C\{^{1}H\}$ NMR spectra of compound **3** (in $CDCl_3$).

Table S1. FET parameters of $(C_{12}H_{25})_2$-iPDT thin-film FETs with SiO_2 gate dielectrics.

sample	μ ($cm^2V^{-1}s^{-1}$)	$\lvert V_{th} \rvert$ (V)	ON/OFF	S (V/decade)	L (μm)	W (μm)
#1	4.9×10^{-1}	67.1	7.9×10^{5}	3.2	150	500
#2	3.3×10^{-1}	64.1	1.1×10^{5}	8.3	250	500
#3	6.1×10^{-1}	62.7	3.2×10^{6}	7.2	300	500
#4	3.1×10^{-1}	61.3	4.5×10^{5}	7.2	300	500
#5	4.2×10^{-1}	65.6	1.7×10^{5}	8.5	350	500
#6	6.6×10^{-1}	51.9	8.2×10^{5}	4.4	450	1000
#7	6.1×10^{-1}	64.4	4.8×10^{5}	8.4	450	1000
#8	8.0×10^{-1}	52.4	4.1×10^{5}	2.7	450	500
#9	2.3×10^{-1}	57.7	1.9×10^{6}	7.9	450	500
#10	3.3×10^{-1}	67.1	1.7×10^{6}	3.8	450	500
**average**	$5(2) \times 10^{-1}$	61(6)	$1(1) \times 10^{6}$	6(2)	-	-

Table S2. FET parameters of $(C_{12}H_{25})_2$-iPDT thin-film FETs with ZrO_2 gate dielectrics.

sample	μ (cm^2 V^{-1} s^{-1})	$\|V_{th}\|$ (V)	ON/OFF	S (V/decade)	L (μm)	W (μm)
#1	3.40	9.57	9.5×10^6	9.1×10^{-1}	100	500
#2	3.58	9.45	1.6×10^6	1.07	200	500
#3	3.79	10.03	1.4×10^4	1.43	250	500
#4	4.02	9.48	5.1×10^5	6.1×10^{-1}	350	500
#5	4.35	9.72	9.1×10^3	1.47	350	500
#6	4.10	9.60	1.3×10^6	1.09	450	1000
#7	5.38	7.84	1.1×10^7	8.1×10^{-1}	450	1000
#8	5.28	8.72	1.3×10^4	1.39	450	500
#9	4.55	13.14	2.4×10^6	7.5×10^{-1}	450	500
#10	4.48	13.01	2.5×10^6	1.12	450	500
#11	3.80	13.02	2.2×10^6	7.9×10^{-1}	450	500
**average**	4.3(6)	10(2)	$3(4) \times 10^6$	1.0(3)	-	-

Table S3. FET parameters of $(C_{12}H_{25})_2$-iPDT thin-film FETs with PZT gate dielectrics.

sample	μ (cm^2 V^{-1} s^{-1})	$\|V_{th}\|$ (V)	ON/OFF	S (V/decade)	L (μm)	W (μm)
#1	3.5	6.3	6.9×10^5	8.6×10^{-1}	250	500
#2	3.1	6.1	6.3×10^6	1.00	250	500
#3	3.2	6.1	1.4×10^6	7.5×10^{-1}	300	500
#4	5.6	6.8	4.6×10^5	7.5×10^{-1}	350	500
#5	5.5	5.8	4.6×10^5	8.4×10^{-1}	350	500
#6	4.0	5.3	8.0×10^5	8.0×10^{-1}	450	500
#7	4.8	8.9	3.2×10^5	1.13	450	500
**average**	4(1)	6(1)	$2(2) \times 10^6$	$9(1) \times 10^{-1}$	-	-
